# Evaluation of Acute and Sub-Acute Toxicity of Aqueous Extracts of *Artemisia afra* Leaves on Brain, Heart and Suprarenal Glands in Swiss Albino Mice

**DOI:** 10.4314/ejhs.v30i6.16

**Published:** 2020-11

**Authors:** Ketema Mekonen, Mekbeb Afework, Eyasu Makonnen, Asfaw Debela, Wondwossen Ergete, Tesfaye Tolessa

**Affiliations:** 1 Department of Anatomy, College of Health Sciences, Debermarkos University, Ethiopia; 2 Department of Anatomy, College of Health Sciences, Addis Ababa University, Ethiopia; 3 Department of Pharmacology, College of Health Sciences, Addis Ababa University, Ethiopia; 4 Center for Innovative Drug Development and Therapeutic Trials for Africa, College of Health Sciences, Addis Ababa University, Ethiopia; 5 Ethiopian Public Health Institute, Ethiopia; 6 Department of Pathology, College of Health Sciences, Addis Ababa University, Ethiopia; 7 Department of physiology, College of Health Sciences, Addis Ababa University, Ethiopia

**Keywords:** A. afra, Toxicity study, histopathology, brain, heart, suprarenal glands, Swiss albino mice

## Abstract

**Background:**

The majority of population rely on traditional medicine as a source of healthcare. Artemisia afra is a plant traditionally used for its medicinal values, including treatment of malaria in many parts of the world. Currently, it is also attracting attention because of a claim that a related species, Artemisia annua, is a remedy for the COVD-19 pandemic. The aim of the present study was to investigate toxic effects of A. afra on brain, heart and suprarenal glands in mice aged 8–12 weeks and weighing 25–30g.

**Methods:**

Leaves of A.afra were collected from Bale National Park, dried under shade, crushed into powder and soaked in distilled water to yield aqueous extract for oral administration. For acute toxicity study, seven treated and one control groups, with 3 female mice each, were used. They were given a single dose of 200mg/kg, 700mg/kg, 1200mg/kg, 2200mg/kg, 3200mg/kg, 4200mg/kg or 5000mg/kg b/wt of the extract. For the sub-acute toxicity study, two treated and one control groups, with 5 female and 5 male mice each, were used. They were daily treated with 600mg/kg or 1800mg/kg b/wt of extract.

**Results:**

LD_50_ was found to be greater than 5000mg/kg indicating that the plant is relatively safe. In the sub-acute study, no signs of toxicity were observed in all treatment groups. On microscopic examination of the brain, heart and suprarenal glands no sign of cellular injury was observed.

**Conclusion:**

The findings of this study suggest that the leaves extract of A. afra is relatively safe in mice.

## Introduction

The genus Artemisia, which belongs to the family of Asteraceae, contains more than 400 species and is widely used in many parts of the world either alone or in combination with other plants as herbal remedy for a variety of human ailments. *A. afra* is a medium-size perennial herb, rarely exceeding 2m high. It is located in Ethiopia Kenya, Zimbabwe, Malawi, Angola and South Africa (1).

Various parts of the plant contain volatile oil, terpenoids, coumarins, acetylenes, scopoletin and flavonoids (2). The volatile oil contains 1, 8-cineole, α-thujone, β-thujone, camphor and borneol, and has definite anti-microbial and anti-oxidative properties. Thujone is known to cause neurotoxicity with different neurological symptoms like dizziness, tremor, convulsion and hallucination (3).

Artemisia species are most commonly used in traditional folk medicine, notably in the treatment of malaria. In Ethiopia, *A. afra* is traditionally used in combination with other herbals as a remedy against headache, eye diseases, ringworm, haematuria and stabbing pain (4). It is also used to treat infertility, febrile illness, common cold, spirit and epilepsy (5).

*A. afra* has recently attracted worldwide attention of researchers for its possible use in the treatment of chronic diseases like diabetes, cardiovascular diseases and cancer. Aqueous extract of *A. afra* has cardioprotective, antihyperlipidemic, antioxidant and antihypertensive activities (6). Its related species *A. annua* is recently claimed from Madagascar to be a remedy for the current COVID-19 pandemic. Ethanol extract of *A. afra* was observed to arrest cell cycle of cancer cells (7). Aqueous extract of *A. afra* was shown to decrease glucose level near normal range and to have antioxidant activity in diabetic rats (8). It was also reported that it has bronchodilation and anti-inflammatory activities (9).

Whilst evidence-based studies indicating the efficacy of herbal remedies are still being unveiled, increasing evidence regarding adverse effects of herbal medicine has highlighted the demand for toxicological studies for herbal products (10). This could also be true for *A. afra* with only limited studies have investigated its toxicity. Acute oral administration of aqueous extract of *A. afra* to mice was non-toxic with LD_50_ of 8960mg/kg (2). The same study showed that chronic oral administration of this extract in rats was relatively safe with minor intermittent diarrhea, salivation and partial hypo-activity. Acute and sub-chronic toxicity studies on the aqueous leaf extract of A. *afra* have also shown no significant sign of toxicity on liver, kidney and some blood parameters in Wistar rats (11). However, studies on the effect of the plant extract on other vital organs are lacking. The aim of the present study was to investigate toxic effects of *A. afra* on brain, heart and suprarenal glands in albno mice.

## Materials and Methods

The study was laboratory based experiment conducted at Addis Ababa University (AAU), College of Health Sciences, Departments of Anatomy and Physiology, and Ethiopian Public Health Institute (EPHI) from September 2014 to July, 2015.

**Collection of plant materials**: *A. afra* was collected from Bale National Park, 400km southeast of Addis Ababa in Oromia regional state during the month of September 2014. The plant was identified by a taxonomist, and a few samples were deposited at the National Herbarium in the College of Natural and Computational Sciences, Addis Ababa University (AAU), with a voucher specimen number of 392/NKI/PHARM.

**Preparation of aqueous leaf extract**: The plant leaves were cleaned, dried in shade, ground to powder (400g) and macerated with distilled water for 2hrs and 30minutes with intermittent agitation by orbital shaker. The supernatant part of agitated materials was decanted and filtered with 0.1 mm^2^ mesh gauze from the un-dissolved portion of the plant. The filtrate was freeze-dried to give 43g (10.75% yield) of crude extract.

**Experimental animals**: The animals used in this study were bred and reared at the animal house of EPHI and transported to AAU, College of Health Sciences, Department of Physiology. Experiments were conducted on 54 healthy adult male and female mice aged 8–12 weeks and weighing 25–30g. Grouping of mice was done randomly. The animals were kept in separate polycarbonate cages and provided with bedding of clean paddy husk. The mice were acclimatized to laboratory conditions for one week prior to experimentation to minimize nonspecific stress (12).

The test substance was administered in single and repeated doses by gavages for acute and sub-acute toxicity studies, respectively. For the acute toxicity study, prior to dosing food but not water was withheld for 3 hours. Following the period of fasting, the animals were weighed, and the dose was calculated according to the body weight for each animal. The test substance dissolved in distilled water was then administered. After the substance was administered, food was withheld for 2 hours (12).

**Acute toxicity study and LD_50_ determination**: Acute toxicity test was done as per the OECD guideline for testing of chemicals 423 (12). It was started with low initial single dose of 200mg/kg. This dose was selected in reference to a previous efficacy study of *A. afra* which showed significant anti-diabetic activity at 200mg/kg in diabetic rats (13). Additional six higher single doses of 700mg/kg, 1200mg/kg, 2200mg/kg, 3200mg/kg, 4200mg/kg and 5000mg/kg were administered. A total of eight groups of mice were used (seven treated and one control) each consisting of 3 female adult albino Swiss mice.

The treated and control groups were observed continuously for 3hrs and then every 24hrs for the next 14 days; and any signs of toxicity and mortality were recorded. The presence or absence of toxic signs like increased motor activity, tremors, ptosis, lacrimation, exophthalmos, piloerection, salivation and depression were observed during the study period. The body weight of each mouse was recorded at the 7^th^ and 14^th^ days. The differences in the body weight were also recorded.

Lethal doses for fifty percent of the mice (LD_50_) for aqueous leaf extracts of *A. afra* were determined using Protocol for LD_50_ determination (12). On the 14^th^ day of treatment, all mice were sacrificed with anesthetic diethyl ether. Comprehensive gross pathological observations were carried out on the brain, heart and suprarenal glands to check for any signs of abnormality and presence of lesions.

**Sub-acute toxicity study**: The study was carried out using 15 female and 15 male mice which were grouped into six groups, 3 groups for the females and another three groups for the males. For both sexes, two groups were given 600mg/kg (low dose) and 1800mg/kg (high dose) of the extract for 28 days, while another group was given vehicle (distilled water). The low dose was selected in reference to an efficacy study of *A. afra* in treatment of malaria which showed 400 mg/kg as effective dose (14). However, this dose was modified to 600 mg/kg based on clinical observation of acute toxicity studies (15). The volume of the extract and distilled water was calculated as 1.5ml/100g and given at constant time between 9:00–10:00 am once a day.

Individual weights of mice were taken shortly before the test substance was administered and weekly thereafter using digital electronic balance. Weight changes were also calculated and recorded. Mice were observed individually once during the first 30 minutes after dosing, four times for the first 4 hours with one hour interval and daily thereafter for 28 days. Clinical observation for morbidity and mortality once a day were recorded (15).

For screening possible neurobehavioral toxicity, in cage and open field observations were done daily. Functional observational battery was employed to assess a wide range of neurobiological functions, including sensory, motor and autonomic components. In the present study, functional observational battery comprising a series of assessments designed to measure motor, sensory and autonomic function was used (16). These included posture, abnormal motor activity (like tremor and fasciculation, convulsion), ease of removal from cage, reactivity to handling, lacrimation anorexia and salivation.

At the end of the test, animals were weighed and then humanely sacrificed anesthetizing with diethyl ether (15). Organs of interest; namely, the brain, heart and suprarenal glands were carefully dissected out and weighed. The relative organ weights of heart and brain were calculated using the animal weight as a denominator, while that of the suprarenal glands' was calculated using the brain as a denominator.

Gross pathological examinations for toxicological lesions, including the presence of dark and white spot and necrosis were done. For the brain, frontal lobes of cerebrum were taken by coronal section at the level of Bregma. Both the right and left suprarenal glands were taken as a sample. The heart was sectioned longitudinally through ventricle and atria from the base to the apex, and the left atria and ventricle were taken for histopathological investigation. Sample tissues were taken and placed in a labeled test tube containing 10% buffered formalin. Fixed tissues were dehydrated and cleared, respectively, in ascending graded series of ethanol and xylene, infiltrated with molten paraffin wax and embedded in paraffin blocks. These were sectioned at a thickness of 5µm using Leica rotary microtome (LEICA RM 2125 RT, Germany). Ribbons of tissue sections were gently collected and placed onto the surface of a water bath heated at 40°C. They were then collected onto gelatin coated glass slides and placed in oven overnight. Sections were deparaffinized by xylene, hydrated through a down series of alcohols, and stained by Harris' hematoxylin. Slides were differentiated by 1% acid alcohol and counter stained in eosin. Stained sections were dehydrated with increasing concentrations of ethyl alcohols and cleared in xylene, mounted using DPX and covered with cover slips.

Stained tissue sections of brain, heart and suprarenal glands were carefully examined under binocular compound light microscope (LEICA DM 750, Germany). Tissue sections from the treated groups were examined for any evidence of histopathological changes by a pathologist with respect to those of the controls blindly. After examination, photomicrographs of selected sample sections of brain, heart and suprarenal glands from both treated and control mice were taken under a magnification of x20 objective by using automated built-in digital photo-camera (EVOS XL, USA).

**Data processing and analysis**: All quantitative data were organized and analyzed using Statistical Package for Social Science (SPSS) version 21 statistical software. The values of body and organ weight including relative organ weights were analyzed, and the results were expressed as mean ±SEM (standard error of mean). Difference between treated and control groups were compared using one-way ANOVA. P-values <0.05 were considered statistically significant.

**Ethical approval**: Ethical approval was obtained from the Research Review Committee of the Department of Anatomy, College of Health Sciences, AAU.

## Results

**Acute toxicity study**: The single oral administration of aqueous extract of *A. afra* in mice did not show any mortality even with the highest dose which was 5000mg/kg. No signs of toxicity were observed at the lower four doses, i.e., 200mg/kg, 700mg/kg, 1200mg/kg and 2200mg/kg. However, signs of mild toxicity like anxiety and piloerection were seen at the doses of 3200, 4200mg/kg and 5000mg/kg. These symptoms gradually disappeared after some wash out periods over two weeks observation. Moreover, there was a gradual increase in body weight of treated mice though not statistically different among the different groups.

**Sub-acute Toxicity**

**Effects of *A. afra* aqueous leaves extract on behavior and body weight of mice**: During the period of 28 days of sub-acute toxicity study, mice treated orally with both low and high doses of the extract showed no noticeable change in their general behavior compared to the control group. There was also no significant difference between the two sexes, except that the males, in both treated and non-treated control groups, were more aggressive compared to the females (Table 1). Moreover, there was no toxicity related death throughout the period of the study.

**Table 1:** Neurobehavioral neurotoxicity evaluation of male and female mice treated with aqueous extract of *A. afra* as compared to the controls during consecutive four weeks of observations

	Male			Female		
Neurologic symptom & sign	600mg/kg	1800mg/kg	Control	600mg/kg	1800mg/kg	Control
Ataxia/abnormal gait	-	-	-	-	-	-
Hunched posture	-	-	-	-	-	-
Pain response (tail pinch)	+	+	+	+	+	+
Convulsions	-	-	-	-	-	-
Depression of open field activity	-	-	-	-	-	-
Anorexia	-	-	-	-	-	-
Irritability/aggressiveness	+	+	+	-	-	-

“+” means behavioral response present. “-” means behavioral response absent. n=5

**Figure 1:**
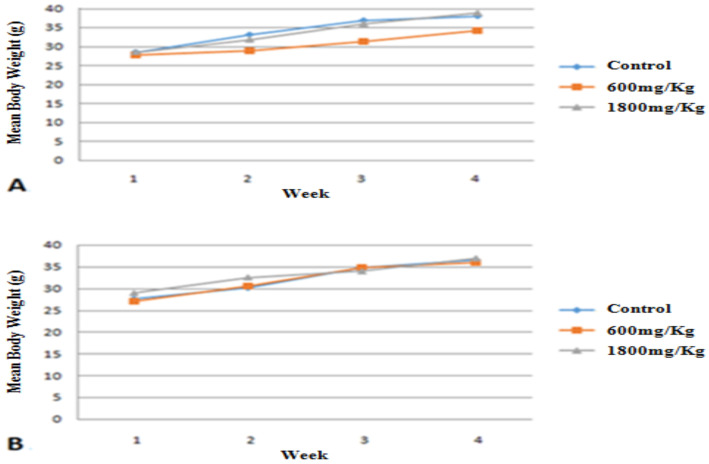
Mean body weight growth between female (A) and male (B) mice treated with 600mg/kg and 1800mg/kg of aqueous leaves extract of A.afra as compared to the controls. Values are m+/-SEM. n=5.

Gradual weight gain was observed in both treated and control groups though not statistically significant during the study period, and no significant difference was also observed between male and female groups (Figure 1).

**Effects of *A. afra* aqueous leaves extract on gross pathology and relative organ weight**: On gross examination of brain, heart and suprarenal glands, no abnormal gross pathological findings like dark and white spot and necrosis were observed in any of the treatment and control groups. There were no significant absolute and relative organ weight changes between treated and control groups in both sexes (Table 2).

**Table 2:** Absolute and relative organ weights of mice orally treated with 600mg/kg and 1800mg/kg of aqueous leaves extract of *A. afra* as compared to the controls

Group	Brain		Heart		Suprarenal glands	
	Absolute weight (mg)	Relative weight (mg)	Absolute weight (mg)	Relative weight (mg)	Absolute weight (mg)	Relative weight (mg)*
**Male**						
**600 mg/kg/day**	0.43±0.02	1.21±0.02	0.15±0.01	0.42±0.02	0.01±0.002	2.32±0.003
**1800 mg/kg/day**	0.44±0.01	1.19±0.03	0.17±0.01	0.46±0.01	0.01±0.004	2.27±0.004
**Control**	0.44±0.2	1.2±0.02	0.15±0.01	0.41±0.01	0.01±0.003	2.27±0.004
**Female**						
**600 mg/kg/day**	0.43±0.02	1.26±0.02	0.15±0.007	0.44±0.01	0.01±0.001	2.32±0.001
**1800 mg/kg/day**	0.45±0.003	1.16±0.02	0.16±0.02	0.41±0.008	0.01±0.002	2.22±0.001
**Control**	0.45±0.002	1.2±0.04	0.15±0.003	0.41±0.004	0.01±0.004	2.22±0.001

* Relative organ is calculated using the weight of brain as a denominator. Each value is expressed as mean ± SEM, n=5 for each group

**Figure 2:**
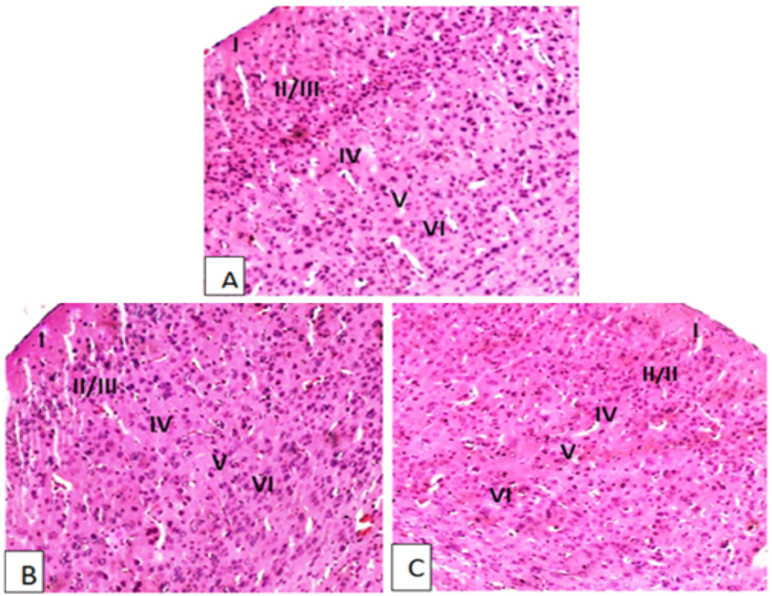
Photomicrographs of H & E stained sections of cerebral cortex from female mice treated with aqueous leaves extract of A. afra at 600mg/kg (B) and 1800mg/kg (C) as compared to the control (A); I = Molecular, II/III = Supragranular Pyramidal, IV = Granular, V = Deep Pyramidal and VI = Polymorphic (Multiform) layers. Magnifications X200

**Effects of aqueous leaves extract on histology of cerebral cortex, heart and suprarenal glands**: Microscopic examination of cerebral cortex of mice treated with 600mg/kg and 1800mg/kg of aqueous leaves extract of *A. afra* indicated no structural disturbance compared to the controls. No signs of individual neuron death and focal lesion such as pyknosis, karyorrhexis and karyolysis and eosinophilic cytoplasm were observed in both male and female mice. In addition, no sign of inflammation like lymphocytic infiltration were observed in male and female mice (Figure 2). The cytoarchitecture of cerebral cortex was identical between treated and control mice.

**Figure 3:**
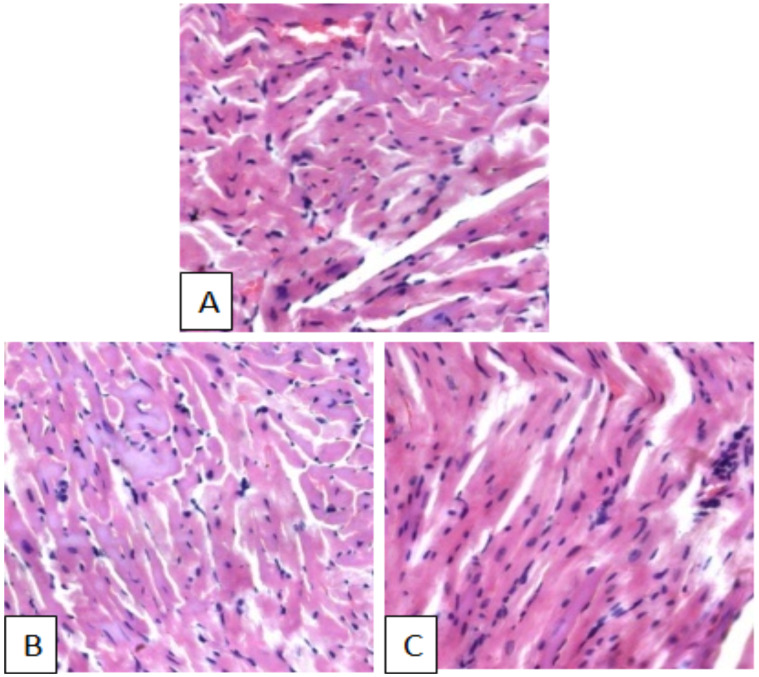
Photomicrographs of H & E stained sections of heart from female mice treated with aqueous leaves extract of A. afraat 600mg/kg (B) and 1800mg/kg (C) as compared to the control (A). Magnifications X300

No architectural difference was observed in microscopic examination of the heart of treated and control female and male mice. In mice treated with both 600mg/kg and 1800mg/kg of the extract, no signs of myocardial cell injury such as pyknosis, karyorrhexis, karyolysis, vacuolation, focal necrosis and fibrosis were observed. Furthermore, no sign of inflammation (leukocytic infiltration) was observed in female and male treated mice groups (Figure 3).

**Figure 4:**
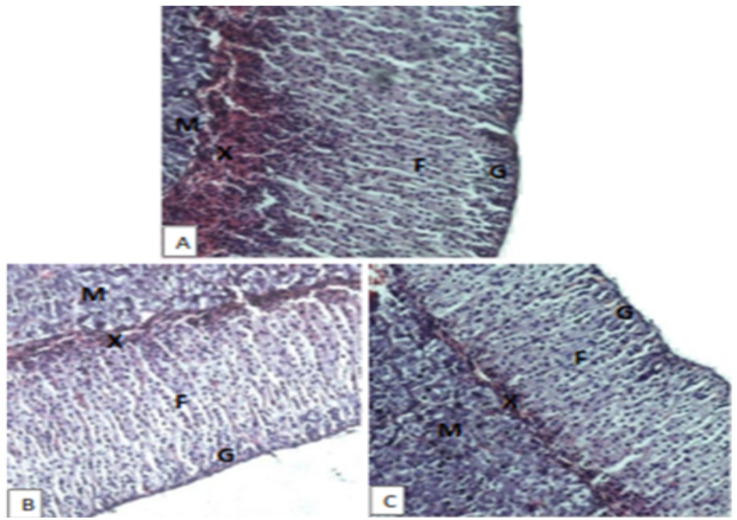
Photomicrographs of H & E stained sections of suprarenal glands from female mice treated with aqueous leaves extract of A. afra at 600mg/kg (B) and 1800mg/kg (C) as compared to the control (A); G = zona golerulosa, F = zona fasciculate, X = x zone and M = adrenal medulla; Magnifications X200

On examination of the sections of suprarenal glands, no signs of toxicity were observed on both cortical and medullary regions. The architecture of both cortex and medulla of mice treated with 600mg/kg and 1800mg/kg of the extract was identical with that of the control. In both treated and control mice, two cortical zones were observed with some mice showing additional zone (X-zone). However, no cortical lesion like degeneration (vacuolar or granular), necrosis or hemorrhage was observed (Figure 4).

## Discussion

Evaluation of the pathological alterations induced in laboratory animals by novel treatment agents represents one of the safety assessments prior to conducting clinical trials. This preliminary assessment represents major contributions to the development of new treatments for humans and animals. Acute toxicity tests provide data on the relative toxicity likely to arise from a single or brief exposure. It is an initial assessment of toxic manifestations induced with the test substance/s (17).

This study showed that the LD_50_ of the aqueous extract of *A. afra* was above 5000mg/kg which is grouped under category 5 or unclassified according to Globally Harmonized Classification System (13). This is in agreement with the findings of previous studies in mice (2) and rats (11) experiments. Oral administrations of the extract up to dose of 2200 mg/kg did not cause any alteration in the behavioral pattern of the mice as compared to the control group. However, mild sign of toxicity was observed at the higher three doses. There was no significant difference in weight gain between treated and control groups. Gross examination of heart, suprarenal glands and brain revealed no treatment-related gross findings at necropsy. The results of acute toxicity test in this study, therefore, indicate that aqueous leaf extract of *A. afra* is tolerated up to the limit of 5000mg/kg body weight as per OECD guidelines (12).

Sub-acute toxicity study provides information on possible health hazards likely to occur on nervous, immune and endocrine systems from repeated exposure over a relatively limited period of time (15). The present subacute toxicity study with oral aqueous extract of *A. afra* on body weight, brain, heart and suprarenal glands showed that no significant behavioral changes were observed between treated and control groups in both sexes. Changes in animals' body weights are usually used as an indicator of toxic effects of test substances (18). On the other hand, increase in animals' body weight could also be related to body fat accumulation rather than to toxicity (19). The non-significant increase in body weight observed in the present study might be attributed to fat accumulation in the body. Evaluation of organ-to-body weight and organ-to-brain weight ratios are also used to assess treatment related effects in toxicological studies. For suprarenal glands, organ-to-brain ratio is more predictive of suprarenal glands weight change than organ-to-body weight ratio (20). No significant changes were observed in absolute and relative organ weights of all groups in the present study (21). These findings are in line with those of previous studies done with other species of the genus Artemisia

In response to injury, a number of changes may occur in neurons and their processes (axons and dendrites) like shrinkage of cell body, pyknosis of the nucleus, disappearance of the nucleolus and loss of Nissl substance with intense eosinophilia of the cytoplasm (red neurons) (22). In the histopathological examination of cerebral cortex, none of these morphological changes was observed which was supported by absence of abnormal behavior and motor activity on cage side observation. This might suggest that the aqueous leaf extract of *A. afra* may not cause toxicity to cerebral cortex of mice.

Because of its high oxidative metabolic need, the heart can be injured by any compounds that interfere with its oxygen supply. On microscopic examination, myocardial damage can take the form of cytoplasmic alterations such as vacillation, pyknotic nucleus, karyrrhexis, and karyolysis in diffuse or localized area (23). In the present study, none of these lesions were observed which is in line with the results of the previous study done on the same plant with different dose (2).

Suprarenal glands are reported to be the most common endocrine organ associated with chemically induced lesions (24). These lesions are more frequent in the zona fasciculata than in the zona glomerulosa. The adrenal cortex produces steroid hormones with a 17-carbon nucleus following a series of hydroxylation reactions that occur in the mitochondria and endoplasmic reticulum. Toxic agents for the adrenal cortex include short-chain aliphatic compounds, lipidosis inducers, amphiphilic compounds, natural and synthetic steroids, and chemicals that affect hydroxylation (25). Morphologic evaluation of cortical lesions provides insight into the sites of inhibition of steroidogenesis. In the present study, no histological degenerative or proliferative lesions of cortex or medulla were observed suggesting the non-toxic effect of the plant on the glands which is in agreement with that of the previous study done in other species of the same genus (21).

In conclusion, the present findings suggest that administration of 600mg/kg and 1800mg/kg of body weight of aqueous leaves extract of *A. afra* in mice for a month is safe.
